# Lightweight Workload Fingerprinting Localization Using Affinity Propagation Clustering and Gaussian Process Regression

**DOI:** 10.3390/s18124267

**Published:** 2018-12-04

**Authors:** Santosh Subedi, Jae-Young Pyun

**Affiliations:** Department of Information and Communication Engineering, Chosun University, Kwangju 501-759, Korea; santoshmsubedi@chosun.kr

**Keywords:** affinity propagation clustering, bluetooth low energy, fingerprinting localization, gaussian process regression

## Abstract

Fingerprinting localization approach is widely used in indoor positioning applications owing to its high reliability. However, the learning procedure of radio signals in fingerprinting is time-consuming and labor-intensive. In this paper, an affinity propagation clustering (APC)-based fingerprinting localization system with Gaussian process regression (GPR) is presented for a practical positioning system with the reduced offline workload and low online computation cost. The proposed system collects sparse received signal strength (RSS) data from the deployed Bluetooth low energy beacons and trains them with the Gaussian process model. As the signal estimation component, GPR predicts not only the mean RSS but also the variance, which indicates the uncertainty of the estimation. The predicted RSS and variance can be employed for probabilistic-based fingerprinting localization. As the clustering component, the APC minimizes the searching space of reference points on the testbed. Consequently, it also helps to reduce the localization estimation error and the computational cost of the positioning system. The proposed method is evaluated through real field deployments. Experimental results show that the proposed method can reduce the offline workload and increase localization accuracy with less computational cost. This method outperforms the existing methods owing to RSS prediction using GPR and RSS clustering using APC.

## 1. Introduction

Indoor location-based service (LBS) has been attracting significant interest in recent times owing to an increase in the number of smart devices and technologies being used. Pervasive applications such as behavior recognition, smart medication, and smart building require accurate position information of the users to yield accurate and timely services [1]. Although the global navigation satellite system is widely used for LBS, its signal cannot penetrate buildings. In addition, indoor environments are often complex owing to the presence of obstacles and environment changes, resulting in signal fluctuation or noise. Nevertheless, high localization accuracy (meter level) is often expected for adequate LBS. Some of the consumer technologies adopted in the development of indoor positioning system (IPS) include Bluetooth low energy (BLE), Wi-Fi, ultra-wideband, visible light wave, and geomagnetism [2,3,4,5,6]. The wireless signal measuring principles in IPS are time of arrival (TOA), time difference of arrival (TDOA), angle of arrival (AOA), and received signal strength (RSS) [7].

Trilateration and fingerprinting localization are used in most IPS studies. Trilateration estimates the position of a tag device by observing its distance from multiple access points (APs). The distance is estimated by employing time-of-arrival between devices and APs or obtaining the RSS from the neighborhood devices. Here, a few nonlinear equations are solved to estimate the 2-D position of the tag device, where three non-collinear and non-collocated APs are located.

Fingerprinting is based on scene analysis that consists of two phases: offline and online. A radio map consisting of both RSS information from the deployed APs and reference point (RP) locations is built in the offline phase and is preserved in a database. Subsequently, it is compared with online-observed RSS data in the online phase to deduce the tag location. Apart from RSS, channel state information [8] and geo-magnetic field strength [9] are used as the location fingerprint. Fingerprinting is a pervasive method governing IPS owing to its high reliability. However, the offline phase of fingerprinting is time-consuming and labor-intensive. Moreover, the radio map needs frequent updates owing to dynamic and unpredictable changes in the radio environment [10]. Hence, the time and effort required to build the radio map act as a tradeoff for the localization reliability of fingerprinting localization. Furthermore, the fingerprinting localization can be realized with probabilistic and deterministic approaches. The probabilistic approach yields localization information by estimating a probability distribution over the reference points, whereas the deterministic approach implements fingerprinting data comparison algorithms to find the estimated position. The probabilistic method of fingerprinting has better localization accuracy than the deterministic method. However, the computational complexity of the probabilistic approach is higher compared to the deterministic approach [11]. Clustering helps to reduce the region of interest from the whole fingerprint database to a subset of it in the online phase, and thus reduces the computational complexity [12].

Many studies have endeavored to reduce the offline workload of fingerprinting localization. A self-guided robot armed with inertial measurement unit sensors is employed in reference [13] to explore the testbed and obtain the RSS data. Although this approach may reduce the human workload, it is not practically feasible. The study in reference [14] combines the traditional fingerprinting with weighted centroid localization (WCL) to yield acceptable location estimation by employing a fewer number of RPs across the testbed. Here, the WCL estimation obtained with nearby beacons is used as coarse localization to determine the distances to the selected k-nearest RPs; further, the estimated distances and the respective coordinate of k-nearest RPs are utilized to run the next WCL operation. Subsequently, the fine localization result is produced. The WK-NN interpolation algorithm is combined with affinity propagation clustering (APC) to reduce the offline workload in [15]. This method employs APC to find trusted neighbors for Wk-NN interpolation. First, the cluster representative is selected and the physical distance between the unknown RPs and the cluster representative RP is calculated. Second, the unknown RPs are divided into corresponding clusters based on minimum distance to cluster representative where signal strength at each unknown RPs is calculated using a Wk-NN interpolation algorithm. Despite the requirement of manually collecting the RSS to begin the algorithm (RSS clustering with APC), this method can be helpful while updating the fingerprint radio map in a timely manner. Another approach to reduce human effort in acquiring the radio map is to use crowdsourcing, machine learning, and a fusion of similarity-based sequence and dead reckoning [16,17,18].

In this paper, we present a probabilistic framework for handling sparse training data through APC-based fingerprinting localization. Additionally, the offline workload is decreased by employing the Gaussian process regression (GPR) in the localization to predict the RSS at locations with no prior measurements. The radio map constructed by using limited training data is further divided into clusters or sub-areas by applying APC. The RSS clustering with APC helps to reduce the localization estimation error and computational complexity. BLE beacon, which consumes less energy, is used in this study as the indoor positioning technology.

The remainder of this paper is organized as follows: Section 2 briefly describes some published literature on fingerprinting localization using GPR and APC. Section 3 introduces a general model of GPR and APC. The proposed positioning method is elaborated in Section 4. Experimental results and discussion are presented in Section 5, and the conclusion is presented in Section 6.

## 2. Related Work

An up-to-date radio map database is required for pragmatic fingerprinting localization applications. Conventionally, it is performed by manually acquiring RSS data at RPs across the testbed. Hence, the offline workload of traditional fingerprinting localization makes the system tedious, time-consuming, and labor-intensive. Whereas in the online phase, the probabilistic approach of fingerprinting positioning has good localization accuracy, but it also has high computational complexity.

The use of regression to predict RSS data with training datasets helps reduce the offline workload of fingerprinting localization. Some representative approaches based on regression or interpolation include polynomial fitting, exponential fitting, and the log model [19]. However, they can predict the mean RSS but not the variance of the estimate. GPR has an advantage of predicting the mean RSS along with the variance, which indicates the uncertainty of the estimation. Hence, GPR is suitable for a probabilistic-based localization method. A GPR-based fingerprinting IPS using indoor Wi-Fi APs is presented in reference [10]. The hyperparameters of the GPR are obtained by using the firefly algorithm to achieve a median localization error of 3 m. It has also been shown that the probabilistic-based localization performs better than the deterministic-based localization. Another example of GPR-based fingerprinting IPS using Wi-Fi is presented in reference [20]. Here, the GPR parameters or the hyperparameters are estimated by using a subspace trust-region method. This work reports that the localization accuracy with a radio map constructed using GPR is higher than that of the Horus fingerprinting method [21]. A GPR plus method using Bluetooth transmitters is proposed in reference [22]. The authors compared their IPS architecture with reference [10] by using the naïve Bayes algorithm to reduce the computation complexity of the IPS.

Clustering helps to minimize the searching space of RPs on the online phase of fingerprinting localization. Some well-known clustering algorithms are K-means, fuzzy c-means, and APC [23,24,25]. An artificial neural network (ANN)-based fingerprinting employing K-means and fuzzy c-means clustering in wireless sensor networks is presented in reference [26]. Here, after dividing the fingerprinting database into clusters, a separate ANN is trained for each cluster by using only those fingerprints that belong to the cluster. Moreover, a prototype signal strength vector is determined for each cluster during clustering and it is compared with online signal strength measurements for final localization estimation. APC has also been widely used in deterministic (Wk-NN) and probabilistic approaches of fingerprinting positioning [27,28,29,30]. All of this literature compares the fingerprint with the cluster heads RSS value in their class-matching methods. In addition to similarity criterion to the RSS of cluster heads, Ref. [27] proposes to use mean of the fingerprints of members of each cluster and reference [28] proposes to use Mahalanobis norm for coarse localization.

Although the GPR and APC are used in many kinds of literature, we find that there is no related literature that combines them to address the real issues of conventional fingerprinting localization like offline workload (labor intensive and time-consuming) and computational complexity. In our study, we use the APC algorithm along with a machine learning approach of fingerprinting localization. The mean RSS and variance are predicted using GPR while using little training data to construct a radio map. RSS clustering is performed on the radio map using APC, i.e., the testbed can be divided into clusters of RPs generated by APC. In the online phase, any exemplar RP is estimated as coarse localization. Subsequently, the RPs grouped under the exemplar RP are used for fine localization. Here, we evaluate two exemplar decision rules depending on the posterior probability and RSS distance as a metric to determine the exemplar RP.

## 3. Background

### 3.1. Gaussian Process Regression

A Gaussian process (GP) is a stochastic process and is an extension of multivariate Gaussian to an infinite-sized collection of real-valued variables. The GP defines a distribution over functions from the view of function space [10,31]. Let us consider an observation model as follows:

(1) y=f(x)+υ,

where υ denotes the independent and identically distributed (i.i.d) Gaussian noise with zero mean and variance $\left(\sigma_{\upsilon }{}^{2}\right)$, which can be summarized as: $\upsilon \;\sim \;N\left(0,\sigma_{\upsilon }{}^{2}\right)$. Here, y and x represent the observed RSS data and RPs location coordinate (input features), respectively.

The mean function, m(x), and covariance function, $k\left(x_{m},x_{n}\right)$, for the latent function, f, can be expressed as

(2) m(x)=E[f(x)]

(3) $$k\left(x_{m},x_{n}\right)=E\left[\left(f\left(x_{m}\right)-m\left(x_{m}\right)\right)\left(f\left(x_{n}\right)-m\left(x_{n}\right)\right)\right],$$

where E(·) represents the expectation operator. For simplification, the mean function can be considered to be zero without loss of generality. The following kernel function is adopted in this study:

(4) $$k\left(x_{m},x_{n}\right)=\sigma_{f}{}^{2}exp\left(-\frac{\left||x_{m}-x_{n}|\right|}{2l^{2}}\right),$$

where $\sigma_{f}{}^{2}$ is the signal variance and l is the length scale parameter. Here, $\theta =[\sigma_{f},l$] are the GPR parameter or the hyperparameter that needs to be optimized [32]. Note that l characterizes the smoothness of the predicted mean RSS. Using (4), the Gramian matrix, $\tilde{K}$, is defined as

(5) $$\tilde{K}=\left[\begin{array}{ccc} k\left(x_{1},x_{1}\right) & \cdots & k\left(x_{1},x_{N}\right)\\ \vdots & \ddots & \vdots \\ k\left(x_{N},x_{1}\right) & \cdots & k\left(x_{N},x_{N}\right) \end{array}\right],$$

where N denotes the total number of RPs across the testbed. Hence, the GP equation is

(6) $$f\left(x\right)\;\sim \;gp\left(m\left(x\right),k\left(x_{m},x_{n}\right)\right).$$

As it is assumed in GP modeling that the data can be represented as a sample from a multivariate Gaussian distribution, it can be inferred that

(7) $$\left[\begin{array}{c} y\\ y^{*} \end{array}\right]\;\sim \;N\left(0,\left[\begin{array}{cc} \tilde{K}{\left(X,X\right)}_{N\times N} & \tilde{K}{\left(X,X^{*}\right)}_{N\times N^{*}}\\ \tilde{K}{\left(X^{*},X\right)}_{N^{*}\times N} & \tilde{K}{\left(X^{*},X^{*}\right)}_{N^{*}\times N^{*}} \end{array}\right]\right),$$

where y and $y^{*}$ are N training and $N^{*}$ test RSS data, respectively. The block covariance matrix represents the size of that particular matrix for N and $N^{*}$. The conditional probability of $y^{*}|y$ can be computed as

(8) $$P\left(\;y^{*}|y\right)\;\sim \;N\left(\tilde{K}\left(X^{*},X\right)\tilde{K}{\left(X,X\right)}^{-1}y,\tilde{K}\left(X^{*},X^{*}\right)-\tilde{K}\left(X^{*},X\right)\tilde{K}{\left(X,X\right)}^{-1}\tilde{K}\left(X,X^{*}\right)\right).$$

Here, the posterior distribution, $P\left(y^{*}|y\right)$ indicates how likely a prediction $y^{*}\;\mathrm{is}$, given the training data y. The prediction $y^{*}$ is the mean of this distribution $\bar{y^{*}}=\;\tilde{K}\left(X^{*},X\right)\tilde{K}{\left(X,X\right)}^{-1}y$, and the uncertainty of the estimation is $var\left(f\left(X\right)\right)=\;\tilde{K}\left(X^{*},X^{*}\right)-\tilde{K}\left(X^{*},X\right)\tilde{K}{\left(X,X\right)}^{-1}\tilde{K}\left(X,X^{*}\right)$. GPR can be used to build a radio map with $\bar{y^{*}}$ and var(f(X)) for locations without prior measurements.

Before estimating the mean RSS and the variance, the GP model needs to be trained by optimizing the unknown hyperparameter vector θ with the training dataset S. The GP hyperparameters can be inferred using procedures like marginal likelihood (ML), cross-validation (CV), and Bayesian optimization [33,34,35]. Use of the ML approach is optimal and computationally efficient when the data truly follows the GP model. Furthermore, ML can be realized with approaches such as Maximum a Posteriori (MAP) estimator and Minimum Mean Square Error (MMSE) estimator. The marginal log-likelihood function for the Gaussian distributed noise is given by [17,31]:

(9) $$\log\;p\left(y|X,\theta \right)=\;-\frac{1}{2}y^{T}{\left[\tilde{K}\left(X,X\right)+\sigma_{v}^{2}I\right]}^{-1}y-\frac{1}{2}\log\left|\tilde{K}\left(X,X\right)+\sigma_{v}^{2}I\right|-\frac{v}{2}log2\pi .$$

The above function is intrinsic to GPR and balances between model complexity and data fit to avoid overly complex models. In this work, we use the limited memory BFGS-B algorithm to solve the optimization problem [36,37].

### 3.2. Affinity Propagation Clustering

APC divides a set of elements into clusters and selects an exemplar to represent each cluster [23]. In IPS, APC algorithm divides the entire testbed into clusters (or radio map into clusters) based on RSS values at the RPs from the deployed APs. The cluster head is searched first on the online phase (coarse localization) based on a specified similarity metric. It helps to reduce the RP space that reduces the computational complexity of the subsequent fine localization and improve the positioning accuracy. APC begins by assigning each element the same chance to become an exemplar, whereas traditional K-means clustering begins by choosing both the number of output clusters and the corresponding random set of initial exemplars [38]. APC outperforms the K-means clustering owing to the initialization-independent property and better selection of cluster heads. Let the radio map ($\psi_{RSS}$) consisting of the predicted mean RSS be:

(10) $$\psi_{RSS}=\left(\begin{array}{ccc} \psi_{1,1} & \begin{array}{cc} \psi_{1,2} & \ldots \end{array} & \psi_{1,N}\\ \begin{array}{c} \psi_{2,1}\\ \vdots \end{array} & \begin{array}{cc} \begin{array}{c} \psi_{2,2}\\ \vdots \end{array} & \begin{array}{c} \ldots \\ \ddots \end{array} \end{array} & \begin{array}{c} \psi_{2,N}\\ \vdots \end{array}\\ \psi_{B,1} & \begin{array}{cc} \psi_{B,2} & \ldots \end{array} & \psi_{B,N} \end{array}\right),$$

where $\psi_{b,j}=\;{\left(\bar{y^{*}}\right)}_{b,j}$ (b = 1, 2, …, B; j = 1, 2, …, N) is the predicted mean RSS of the $b^{th}$ beacon ($AP_{b}$) signal at the $j^{th}$ RP ($RP_{j}$).

APC uses a pairwise similarity $sim^{CL}\left({\mathrm{RP}}_{i},{\mathrm{RP}}_{j}\right)$ (for i≠j) to describe the fitness of ${\mathrm{RP}}_{j}$ to be selected as the exemplar with respect to ${\mathrm{RP}}_{i}$. Here, the subscript CL indicates that the similarity is evaluated during the clustering step of operation. The pairwise similarity is defined by squared Euclidean distance as follows:

(11) $$sim^{CL}\left({\mathrm{RP}}_{i},{\mathrm{RP}}_{j}\right)=-\|{\vec{\psi }}_{i}-{\vec{\psi }}_{j}\|{}^{2},\forall i,j∊\left\{1,2,\ldots ,N\right\}$$

where ${\vec{\psi }}_{j}={\left[\psi_{1,j},\;\psi_{2,j},\;\ldots \ldots ,\psi_{B,j}\right]}^{T}$.

The self-similarity or preference (Pref) is defined as

(12) $$Pref=median\left\{sim^{CL}\left({\mathrm{RP}}_{i},{\mathrm{RP}}_{j}\right)\right\},\forall i,j∊\left\{1,2,\ldots ,N\right\},\;i\neq j.$$

Two types of messages, namely responsibility, and availability, are transmitted among the RPs for APC. The responsibility message ($r\left({\mathrm{RP}}_{i},{\mathrm{RP}}_{j}\right)$) contains the information about the clustering head whereas the availability message ($a\left({\mathrm{RP}}_{i},{\mathrm{RP}}_{j}\right)$) provides the attachment relations between the RPs and clusters. Both the messages are iteratively updated according to the following relationships:

(13) r(RPi,RPj)= simCL(RPi,RPj)−maxj′≠j{a(RPi,RPj′)+simCL(RPi,RPj′)}

(14) $$a\left({\mathrm{RP}}_{i},{\mathrm{RP}}_{j}\right)=min\left\{0,r\left({\mathrm{RP}}_{j},{\mathrm{RP}}_{j}\right)+\underset{i^{\prime }\neq i,j}{\sum }max\left\{0,r\left({\mathrm{RP}}_{i^{\prime }},{\mathrm{RP}}_{j}\right)\right\}\right\}.$$

A damping factor (γ)∊[0.5,1) is introduced to avoid the possible ringing oscillations while updating the messages (13) and (14). The resulting values of responsibility and availability are:

(15) $$r_{t}\left({\mathrm{RP}}_{i},{\mathrm{RP}}_{j}\right)=\;\gamma *r_{t-1}\left({\mathrm{RP}}_{i},{\mathrm{RP}}_{j}\right)+\left(1-\gamma \right)*r_{t}\left({\mathrm{RP}}_{i},{\mathrm{RP}}_{j}\right),\;a_{t}\left({\mathrm{RP}}_{i},{\mathrm{RP}}_{j}\right)=\gamma *a_{t-1}\left({\mathrm{RP}}_{i},{\mathrm{RP}}_{j}\right)+\left(1-\gamma \right)*a_{t}\left({\mathrm{RP}}_{i},{\mathrm{RP}}_{j}\right)$$

In (15), $r_{t}\left({\mathrm{RP}}_{i},{\mathrm{RP}}_{j}\right)$ and $a_{t}\left({\mathrm{RP}}_{i},{\mathrm{RP}}_{j}\right)$ corresponds to the value of responsibility and availability of the current iteration, respectively. Similarly, $r_{t-1}\left({\mathrm{RP}}_{i},{\mathrm{RP}}_{j}\right)$ and $a_{t-1}\left({\mathrm{RP}}_{i},{\mathrm{RP}}_{j}\right)$ is the value of responsibility and value of availability of the last iteration. Here, greater the sum of $r_{t}\left({\mathrm{RP}}_{i},{\mathrm{RP}}_{j}\right)$ and $a_{t}s\left({\mathrm{RP}}_{i},{\mathrm{RP}}_{j}\right)$, the greater the probability of ${\mathrm{RP}}_{j}$ be the cluster head for the ${\mathrm{RP}}_{i}$. The APC connects all the points in the large space and makes each node (RP in our case) a potential exemplar. APC updates the responsibility and availability message iteratively until it converges and yields clusters and its members finally.

## 4. GPR/APC-Based Fingerprinting Localization

The proposed APC-based fingerprinting localization method adopting GPR has two phases of operations, namely offline and online. In the offline phase, we collect the training data from a few sparse RPs that are a subset of the total RPs on the testbed. The testbed is divided into N uniform grids and each grid center represents an RP. Hence, the testbed has N RPs and the RSS data of B beacons (B APs) are acquired at $r^{th}$ RP (r∈{1, 2,…,N}) q times, that is

$$AP_{r}=\left\{ap_{r,1},\;ap_{r,2},\ldots \ldots \ldots ,ap_{r,B}\right\},$$

where

$$ap_{r,i}=Avg\left(ap_{r,i}^{1},ap_{r,i}^{2},\ldots \ldots ,ap_{r,i}^{q}\right).$$

The observed online RSS data at the unknown location inside the testbed can be represented as follows:

$$AP_{*}=\left\{ap_{*,1},\;ap_{*,2},\ldots \ldots \ldots ,ap_{*,B}\right\}.$$

**Figure 1:**
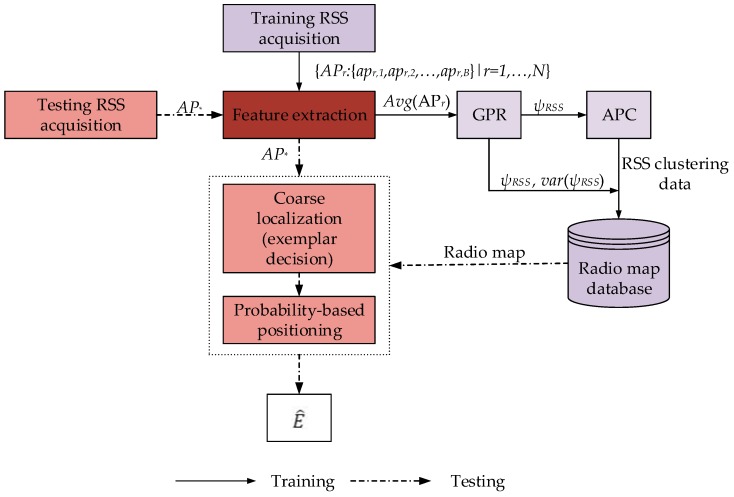
Framework of the proposed APC-based fingerprinting localization adopting GPR.

The framework of the proposed localization system is illustrated in Figure 1. First, training data are collected from sparse RPs (S) that are a subset to N (S<N). Here, for the stability of the randomly fluctuating RSS data, a time average of RSS data is considered. Subsequently, GPR is employed to build the posterior mean RSS and variance at each of the RPs on the testbed. Later on, RSS clustering of the predicted RSS is performed using APC. The predicted RSS mean and variance from the GPR and the RSS clustering with APC are stored in the database for future reference.

Each RP is assigned a probability computed as the probability of $RP_{m}$, given the online RSS observation vector $AP_{*}$. The posterior probability is given as

(16) $$Pr\left(RP_{m}|AP_{*}\right)\;=\;\frac{Pr\left(AP_{*}|RP_{m}\right)\;Pr\left(RP_{m}\right)}{\sum_{n=1}^{N}Pr\left(AP_{*}|RP_{n}\right)\;Pr\left(RP_{n}\right)},$$

where $Pr\left(AP_{*}|{\mathrm{RP}}_{m}\right)$ is the likelihood defined by the following relationship:

(17) $$Pr\left(AP_{*}|RP_{m}\right)=\overset{B}{\underset{b=1}{\prod }}\frac{1}{\sqrt{2\pi \sigma_{b}{}^{2}}}exp\left(-\frac{{\left|AP_{b}-\mu_{b}\right|}^{2}}{2\sigma_{b}{}^{2}}\right).$$

Here,$\;\mu_{b}$ and $\sigma_{b}{}^{2}$ are the predicted mean RSS and its variance of beacon b signal at the RP location, respectively. We define ∁ and $N^{\prime }$ as the set of cluster heads and the number of RPs grouped under a cluster head, respectively. Note that $N^{\prime }$ may vary with the cluster, where $N^{\prime }$ < N. First, coarse localization is performed to determine the exemplar RP. In our proposed APC localization system, the exemplar RP $\left(RP_{e}\right)$ can be determined in either of the following ways:

(18) $$RP_{e}=argmax_{m}\;\left[Pr\left(RP_{m}|AP_{*}\right)\right],\;m\in ∁$$

(19) $$RP_{e}=argmin_{m\in ∁}\vec{\psi_{r}}-{\vec{\psi_{m}}}^{2},\;\vec{\psi_{r}}={\left[\psi_{1,r},\;\psi_{2,r},\;\ldots \ldots ,\psi_{B,r}\right]}^{T}$$

In reference (18), given the online RSS, any RP in ∁ that has the largest posterior probability is determined as the cluster head, whereas in (19), the RP in ∁ with the least RSS distance to the online-acquired RSS data is selected as the cluster head.

Second, the final localization estimation is obtained as a weighted sum of $N^{\prime }$ *RPs* with their respective probabilities.

(20) $$\hat{E}=\overset{N^{\prime }}{\underset{i=1}{\sum }}Pr\left(RP_{i}|AP_{*}\right)\times RP_{i}$$

## 5. Experimental Results

**Figure 2:**
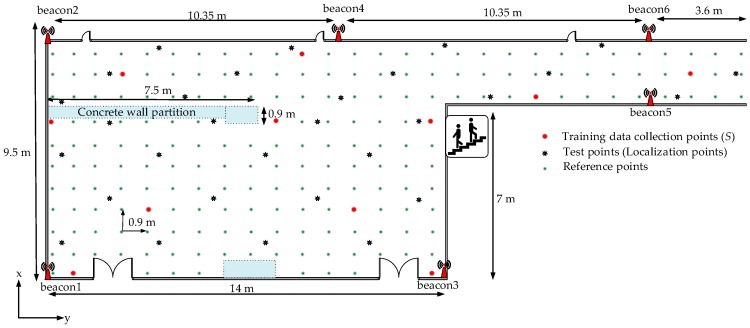
Graphical representation of the testbed (A hallway at the IT building in Chosun University, Korea).

The experimental results are presented in this section. The offline measurements were obtained on the third floor of the IT building, Chosun University, South Korea. The testbed considered is an academic environment with frequent movement of people along the hallways. Six BLE beacons were deployed to cover the whole range of the testbed area. All the beacons were set to have an advertisement interval of 300 ms and beacon transmission power of +4 dBm. The testbed area with the deployed beacons is shown in Figure 2. The testbed area was divided into uniform grids with side measurements of 0.9 m to form 203 measurement locations. An iOS application on iPhone-6S was used to record the RSS data from six beacons. The training data were measured at 11 randomly selected sparse locations (S=11) where 10 time samples of RSS in four different directions (total of 40 samples) were measured at each measurement location. For the locations with undetected beacons or null reading from the beacons, a default RSS value considered −95 dBm in our work was used as invalid data [10,27].

**Figure 3:**
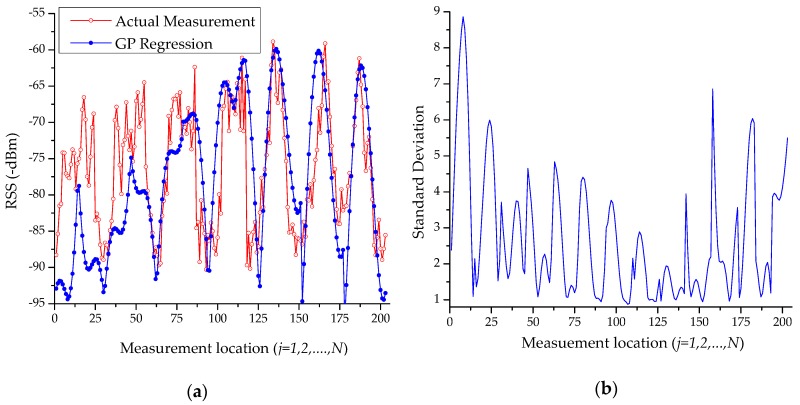
Estimation of $beacon_{4}$ signal with GPR (**a**) predicted RSS, (**b**) the corresponding standard deviation at the measurement locations.

**Figure 4:**
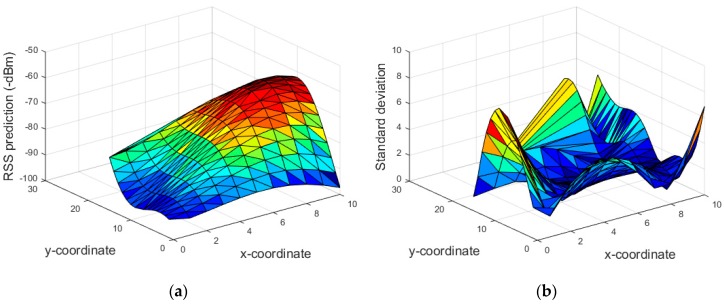
Surface plot of $beacon_{4}$ signal (**a**) predicted RSS, (**b**) corresponding standard deviation.

Figure 3a presents the predicted mean RSS as well as the manually measured RSS at the testbed. It is observed that the predicted RSS is almost similar to the real measured data; in particular, the average difference between the measured and predicted RSS data is 6.50 dBm. Figure 3b presents the corresponding standard deviation of the predicted mean RSS. Figure 4a shows the surface plot of the predicted RSS of $beacon_{4}$ on the testbed. The surface in red depicts the region on the testbed close to the $beacon_{4}$ location in Figure 2. Similarly, Figure 4b shows the surface plot of its standard deviation. Here, the surface in blue indicates that measurement data are available in the surroundings, whereas the red/yellow surface indicates the lack of measurement data around it.

**Figure 5:**
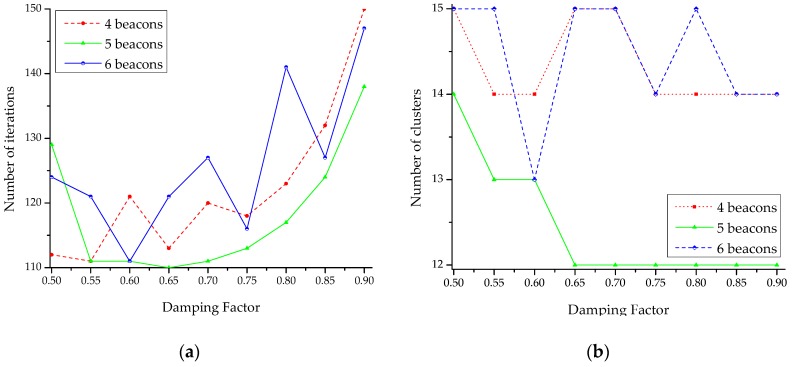
Effect of damping factor (γ) in APC (**a**) the number of clustering operation iterations, (**b**) the number of clusters with respect to the damping factor.

After the GPR operation, the predicted mean RSS was fed to the APC for RSS clustering. While updating the responsibility and availability messages in APC, we evaluated the APC operation by varying the damping factor (γ)∊[0.5, 0.9] introduced in (15). The obtained clustering result is presented in Figure 5.

**Figure 6:**
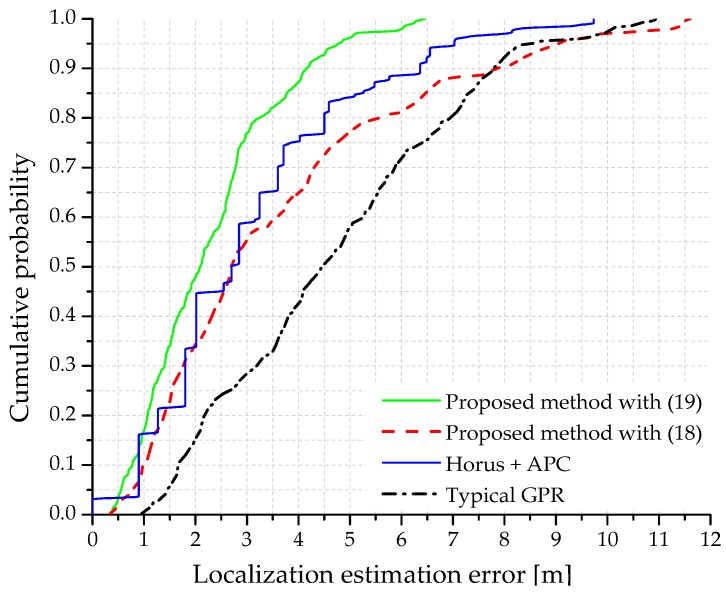
Cumulative probability of the localization estimation error.

We evaluated the localization methods under the deployment of different numbers of beacons on the testbed, i.e., 4, 5, and 6 beacons. For the deployment scenarios of 4 and 5 beacons in the testbed, {$beacon_{4},\;beacon_{5}$} and {$beacon_{5}$} were removed from the testbed, respectively (see Figure 2). As shown in Figure 5, the damping factor with *γ* = 0.6 yields the APC result with the least number of iterations or the least computational cost. We used this value for the localization estimation in our work. The cumulative probability of the localization estimation error in the testbed area is shown in Figure 6. Both the decision rules for exemplar estimation, (18) and (19), presented in Section 4 are considered. At each point of 30 uniformly distributed test locations on the testbed, 15 samples of localization estimation were recorded. We compared our method with a typical GPR-based probabilistic method [20] and Horus method [39]. The Horus method estimates the position of the tag device at the location on the testbed with the largest posterior probability through the Bayesian interference [40]. Moreover, the Horus method employs a clustering module where any cluster is a set of RPs sharing a common set of access points (Wi-Fi). In this study, we applied APC to the manually measured RSS data in the Horus method, where (19) determined the cluster head. In Figure 6, only the Horus method uses the manually constructed radio map data whereas the other methods use the predicted RSS data. Similarly, all the methods under study except the typical GPR employ APC for localization estimation.

As shown in Figure 6, in the proposed method utilizing the RSS distance as the exemplar decision rule, there is a probability of 48% that the localization error is below 2 m. Similarly, in the typical GPR and Horus methods, the corresponding probabilities are 15% and 34%, respectively. The proposed method utilizing the RSS distance shown in (19) as the exemplar decision rule performed better than the method using the largest posterior probability in (18). We recommend the use of (19) for the proposed method, whereas (18) can be used when (19) is not available. Note that approximately 7.5% of the localization estimation error is above 4.5 m in the proposed method owing to the following reasons:

- Faulty exemplar estimation in the online phase: Owing to the fluctuations in the received RSS, the system may lead to the estimation of an RP as an exemplar that is physically far from the real position of the tag device. As the RPs grouped under the cluster head or exemplar are responsible for the final localization estimation, the wrong estimation of exemplar eventually adds some localization estimation error.
- Outliers in the offline phase: The APC algorithm might lead to an RP that belongs to a cluster, but is physically far from the cluster head [30]. This problem can be solved by considering the benefit of the known position of each RP. Here, each outlier RP can be forced to join the cluster characterized by the exemplar at the minimum distance from the outlier itself. This process helps reduce the localization error during the localization operation.

**Figure 7:**
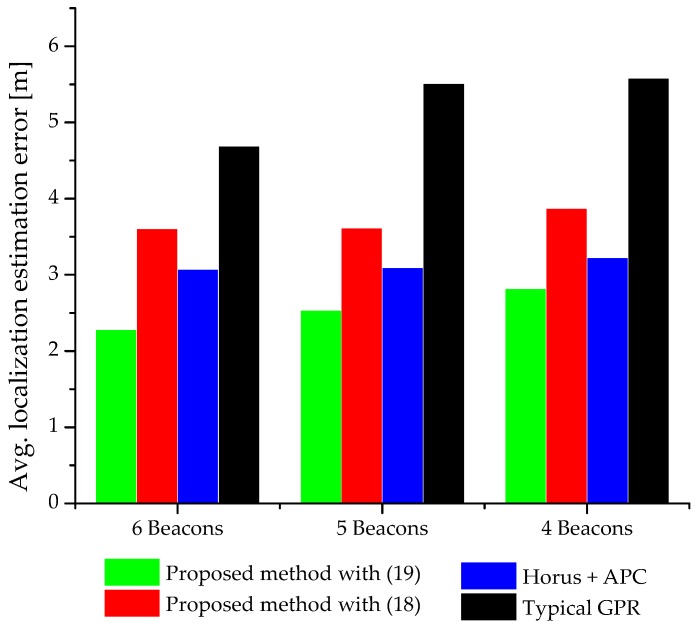
Average localization error from different positioning methods at different beacon density scenarios.

The localization estimation error of the typical GPR method is larger than that of the Horus (with APC) method. However, recording the RSS distribution at RPs in the Horus method is time-consuming. The localization accuracy of the typical GPR method can be improved by increasing the number of training data (S>11 in this work) [10]. However, increasing the size of training data would also increase the offline workload. The average localization estimation error of various positioning methods with different numbers of beacons deployed on the testbed is presented in Figure 7.

Figure 7 shows the impact of different beacon densities on the positioning result. As expected, decreasing the beacon density on the testbed has an inverse effect on the positioning result. The proposed method addresses the real issues of conventional fingerprinting localization to realize a practical IPS. The time consuming and labor-intensive problems of offline phase in conventional fingerprinting are minimized by using GPR to predict the mean RSS and standard deviation at RPs with no prior measurements. For example, we train our GP model using training data from 11 measurement locations to populate the radio map for the entire testbed (203 RPs). Similarly, variously published literature has shown that the computational complexity of the probabilistic approaches of fingerprinting localization is high and the localization accuracy of the deterministic approaches is low. The proposed method solves the computational complexity problem of the probabilistic method using APC.

### Computational Complexity

Finally, we discuss the complexity of our proposed method and the conventional probabilistic fingerprinting method. For the conventional probabilistic fingerprinting method, its complexity is O(NB), where N is the total number of fingerprints to be compared and B is the total number of APs (beacons) in a given indoor environment, respectively. Here, the computational complexity grows with N. The coarse localization stage (cluster head determination stage of online phase) of the proposed method reduces the area of interest from N RPs into $N^{\prime }$ RPs ($N^{\prime }$ is number of RPs grouped under a cluster head by APC and $N^{\prime }$<<N). Hence, the total number of fingerprints to be compared for fine localization is $N^{\prime }$, the complexity of the proposed method becomes $O\left(N^{\prime }B\right)$. Therefore, it can be seen that APC minimizes the searching space of RPs and reduces the computational complexity.

## 6. Conclusions

Despite the reliability of fingerprinting localization in terms of localization accuracy, the main issue with this method is the offline workload that is both tedious and time-consuming. We have employed GPR to overcome the labor-intensive task of the traditional fingerprinting method. In addition, we have performed clustering on the predicted RSS data to divide the whole testbed into many subsections or clusters, which helps to minimize the computational cost of online positioning. The choice of GPR for regression is due to its property of yielding both the mean RSS and the variance, which can be used for probabilistic fingerprinting. APC is selected for clustering owing to its initialization-independent property and better selection of cluster heads compared with K-means clustering.

We used BLE beacons to implement the proposed method considering that they are embedded in many current smartphones and consume less energy than other technologies. We evaluated two different cluster head estimation approaches in the online phase. The experimental result on our testbed shows that the RSS-distance-based exemplar decision method performs better for estimating the cluster head. The positioning result of the proposed method is better than that of the existing methods.
